# Body composition is associated with disease aetiology and prognosis in patients undergoing resection of intrahepatic cholangiocarcinoma

**DOI:** 10.1002/cam4.6374

**Published:** 2023-07-26

**Authors:** Isabella Lurje, Deniz Uluk, Sandra Pavicevic, Minh Duc Phan, Dennis Eurich, Uli Fehrenbach, Dominik Geisel, Timo Alexander Auer, Uwe Pelzer, Dominik Paul Modest, Nathanael Raschzok, Igor Maximilian Sauer, Wenzel Schöning, Frank Tacke, Johann Pratschke, Georg Lurje

**Affiliations:** ^1^ Department of Hepatology and Gastroenterology, Campus Charité Mitte | Campus Virchow‐Klinikum Charité – Universitätsmedizin Berlin Berlin Germany; ^2^ Department of Surgery, Campus Charité Mitte | Campus Virchow‐Klinikum Charité – Universitätsmedizin Berlin Berlin Germany; ^3^ Department of Radiology Charité – Universitätsmedizin Berlin Berlin Germany; ^4^ Department of Hematology, Oncology, and Tumor Immunology Charité – Universitätsmedizin Berlin Berlin Germany

**Keywords:** liver cancer, MAFLD, NAFLD, visceral obesity

## Abstract

**Background:**

Body composition alterations are frequent in patients with cancer or chronic liver disease, but their prognostic value remains unclear in many cancer entities.

**Objective:**

We investigated the impact of disease aetiology and body composition after surgery for intrahepatic cholangiocarcinoma (iCCA), a rare and understudied cancer entity in European and North American cohorts.

**Methods:**

Computer tomography‐based assessment of body composition at the level of the third lumbar vertebra was performed in 173 patients undergoing curative‐intent liver resection for iCCA at the Department of Surgery, Charité – Universitätsmedizin Berlin. Muscle mass and ‐composition as well as subcutaneous and visceral adipose tissue quantity were determined semi‐automatically. (Secondary) sarcopenia, sarcopenic obesity, myosteatosis, visceral and subcutaneous obesity were correlated to clinicopathological data.

**Results:**

Sarcopenia was associated with post‐operative morbidity (intraoperative transfusions [*p* = 0.027], Clavien–Dindo ≥ IIIb complications [*p* = 0.030], post‐operative comprehensive complication index, CCI [*p* < 0.001]). Inferior overall survival was noted in patients with myosteatosis (33 vs. 23 months, *p* = 0.020).

Fifty‐eight patients (34%) had metabolic (dysfunction)‐associated fatty liver disease (MAFLD) and had a significantly higher incidence of sarcopenic (*p* = 0.006), visceral (*p* < 0.001) and subcutaneous obesity (*p* < 0.001). Patients with MAFLD had longer time‐to‐recurrence (median: 38 vs. 12 months, *p* = 0.025, log‐rank test). Multivariable cox regression analysis confirmed only clinical, and not body, composition parameters (age > 65, fresh frozen plasma transfusions) as independently prognostic for overall survival.

**Conclusion:**

This study evidenced a high prevalence of MAFLD in iCCA, suggesting its potential contribution to disease aetiology. Alterations of muscle mass and adipose tissue were more frequent in patients with MAFLD.

## Graphical abstract

Alterations of muscle mass and adipose tissue hold univariable prognostic value in patients with intrahepatic cholangiocarcinoma. While being associated with body composition phenotypes regarded as pathological, fatty liver disease may be associated with longer time to recurrence in patients undergoing surgery for intrahepatic cholangiocarcinoma.

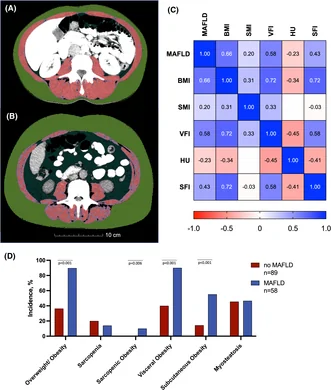

## 1 INTRODUCTION

Intrahepatic cholangiocarcinoma (iCCA) is the second most common primary liver cancer after hepatocellular carcinoma (HCC).^1^ The incidence and mortality of iCCA is rising in western countries, despite an overall declining cancer mortality over the last three decades.^2^ While many cases of iCCA in Europe arise sporadically, known risk factors include chronic inflammation, cholelithiasis and cirrhosis, while liver fluke infections strongly contribute to iCCA incidence in Asian regions.^3^ Generally, the aetiology and disease course of iCCA are understudied in western cohorts.

Recently, obesity and non‐alcoholic fatty liver disease (NAFLD), or, according to recent pathophysiological classifications, metabolic (dysfunction)‐associated fatty liver disease (MAFLD), have been recognized as an iCCA aetiology and suggested as an adverse prognostic factor for overall survival.^4^, ^5^ MAFLD is estimated to affect 25% of the global population and constitutes an important aetiology of end‐stage and malignant liver disease.^6^ A rising incidence and disease severity is projected for the population worldwide, including the USA, China and Europe.^7^ Patients with fatty liver disease furthermore carry a high overall risk of adverse cardiovascular events, other oncological diseases and musculoskeletal disorders.

Body composition—the quantity of skeletal muscle and the quantity and distribution of adipose tissue—of patients with obesity fulfilling non‐alcoholic steatohepatitis (NASH) criteria was characterized by various groups, suggesting that patients typically have a concomitant increase of both adipose tissue and skeletal muscle mass.^8^ It is currently unclear whether these observations can be translated into oncological settings, where the typical phenotype of cachexia and sarcopenia is the most frequently described alteration. Our group recently reported that sarcopenic obesity may hold value for overall survival in iCCA in a different patient cohort from a German tertiary centre.^9^ This publication, despite a lower sample size, showed a high prevalence of body composition pathologies in patients with iCCA and perihilar CCA.^9^

In gastrointestinal malignancies, an association of body composition with metabolic function, systemic inflammatory processes and overall prognosis has been suggested previously.^10^, ^11^ Consequently, nutritional and rehabilitative strategies to maintain and increase muscle mass have been incorporated into guidelines for these patients.^12^ It is currently unclear whether patients suffering from MAFLD‐iCCA show alterations in their body composition, and whether these changes affect disease prognosis. We therefore aimed to delineate prognostic roles of disease aetiology and body composition in iCCA. Furthermore, we examined the relationship between underlying liver disease and pathologies in the computed tomography (CT)‐based composition and quantity of muscle and adipose tissue distribution in a European cohort of surgically treated patients with iCCA.

## 2 METHODS

All consecutive patients undergoing curative‐intent hepatectomy for iCCA between March 2010 and December 2020 at the Department of Surgery, Charité – Universitätsmedizin Berlin were retrospectively evaluated for study inclusion. Inclusion criteria were defined as, (a) pathological and radiological diagnosis of iCCA under the exclusion of perihilar and distal tumour subtypes and mixed iCCA‐HCC, (b) available CT scans including the third lumbal vertebra within 3 months prior to operation. Patients with only other available imaging modalities, such as magnetic resonance imaging (MRI)‐based abdominal staging, were not included in this study.

This study was approved by the ethics committee of the Charité – Universitätsmedizin Berlin (EA1/105/21) and conducted in accordance with the Declaration of Helsinki and the good clinical practice (ICH‐GCP) guidelines. Informed consent was waived in agreement with the ethics committee due to retrospective, pseudonymized study design and analysis of available clinical data. Clinical data were retrieved from patients' records and from a prospectively managed institutional database. Recurrence and survival data were obtained from the Charité outpatient clinic and from local outpatient hepatologists and oncologists.

Post‐hepatectomy liver failure (PHLF), as a potential result of a diminished overall energy reserve, was defined as based on post‐operative (Day 5) international normalized ratio (INR) together with hyperbilirubinemia as previously described.^13^ The presence of MAFLD, as opposed to just the presence of steatosis in our exploratory analysis, was defined based on the pathophysiology‐centred 2020 consensus statement (any presence of pathology‐proven hepatic steatosis plus Type 2 diabetes or a body mass index [BMI] ≥25 kg/m^2^ or the presence of more than two metabolic abnormalities).^14^ Histological steatosis was routinely assessed on Haematoxylin‐Eosin staining and reported in the non‐tumourous area of the resected specimen by a surgical pathologist. In contrast to previous NAFLD criteria, this pathophysiology‐centred approach uses positive inclusion criteria and does not rely on patient‐reported alcohol consumption,^14^ consistent with the paradigm shift towards (a) relevant alcohol consumption being often reported inaccurately (and not routinely investigated prior to oncological surgery as opposed to liver transplant recipients with unclear consumption status) (b) the copresence and thus the substantial etiological overlap of the metabolic syndrome and relevant alcohol consumption^15^ (c) the more accurate reflection on the pathophysiological aspects of the metabolic syndrome as drivers of liver disease.^16^

### 2.1 Image and clinical data analysis

As previously described,^9^, ^17^ an axial CT image at the level of the third lumbar vertebra from the most recent CT image before surgery was analyzed semi‐automatically with 3D Slicer^18^ and the Workstation SlicerCIP extension, body composition module (version 4.10.2). Attenuation values from −29 to 150 Hounsfield units (HU) defined skeletal muscle.^19^ The spinal muscle area (SMA) included the psoas major, spinal (erector spinae, quadratus lumborum), transversus abdominis, external and internal oblique, and rectus abdominis muscles. Attenuation values from −150 to −50 HU indicated visceral adipose tissue, while −190 to −30 HU defined subcutaneous adipose tissue (subcutaneous fat area [SFA]). Skeletal muscle radiation attenuation (SM‐RA) in HU within the muscle area was recorded to assess myosteatosis. Muscle and adipose tissue indices (skeletal muscle index [SMI]; subcutaneous fat index [SFI]) were calculated by normalizing the SMA and SFA to the patients' height (area[cm]^2^/height[m]^2^). The same trained investigator performed the segmentation analysis while being blinded to patients' outcomes (MDP).

Cut‐offs for body composition pathologies were derived from large multicentric oncological cohorts to avoid overfitting to the present dataset.^20^ Primary sarcopenia is defined as low muscle mass and low muscle strength,^21^ while our assessment of sarcopenia relied only on the definition of low muscle mass with the following sex‐specific cut‐off: SMI <52.4 cm^2^/m^2^ for men and <38.5 cm^2^/m^2^ for women.^22^ Hereafter, this CT‐based diagnosis will be referred to as ‘sarcopenia’. The cut‐off for myosteatosis was <41 HU if the BMI was <25 kg/m^2^ and <33 HU for patients whose BMI equalled or exceeded 25 kg/m^2^.^20^ The adaptation to BMI is based on the fact that an accumulation of inter‐ and intramyocellular fat is significantly dependent on the overall amount of body fat and can only be considered pathological in the context of BMI. A visceral fat area (VFA) exceeding 100 cm^2^ indicated visceral obesity,^19^ while the SFI was dichotomized at the upper tertile of the cohort (71.89 cm^2^/m^2^).^23^ Sarcopenic obesity was defined as sarcopenia plus a simultaneous BMI ≥25 kg/m^2^.^9^

### 2.2 Statistical analysis

The primary end point of the present study was oncological and overall survival in patients with iCCA depending on their body composition. The SPSS Statistics 24 software (IBM Corp.) was used for statistical analyses and GraphPad Prism 9 (GraphPad Software) was used for visualization of correlation matrices. Categorical variables were presented as number (frequency, %) and compared with the Mann–Whitney *U* test, while continuous variables (normally distributed) were displayed as mean ± standard deviation and compared with the Pearson's chi‐square test. Non‐normally distributed data were presented as median and range. For comparisons between 3 or more variables, the ANOVA with post hoc Bonferroni correction was used for continuous variables, and the Kruskal–Wallis test for categorical variables. Spearman *r* correlation was calculated and plotted for the association of MAFLD and body composition parameters. A two‐sided *p*‐value of ≤0.05 was regarded as statistically significant, unless corrected with the Bonferroni method. Median time to recurrence (TTR) and overall survival (OS) were presented with 95% confidence intervals (CI). For TTR, the time between operation and recurrence was calculated, and patients were censored if they died or were lost to follow‐up. For OS, the time from operation to death (from any cause) was calculated, and patients were censored at the time of their loss to follow‐up. Survival differences between groups were compared using the Kaplan–Meier curves and the log‐rank test, hazard ratios (HR) were calculated with univariable and multivariable survival analysis. Statistically significant covariates in univariable analysis that are stable over time, and under exclusion of parameters with suspected collinearity were included in the multivariable analyses.

## 3 RESULTS

Of 236 patients with iCCA who were operated at the Department of Surgery, Charité – Universitätsmedizin Berlin within the study period, 173 patients (73%) met the inclusion criteria. The remaining patients either underwent MRI preoperatively or CT images were unavailable for body composition analyses (Figure S1). The study cohort was composed of 87 men (50%) and 86 women (50%) with a mean age of 64 years.

Based on the medical history, tumour aetiology was unknown in most cases (149/173, 86%), while 16 patients reported elevated alcohol consumption (9%), 2 patients (1%) had a previously diagnosed primary sclerosing cholangitis and 6 patients (4%) had chronic viral hepatitis. Histological data on underlying liver disease were available in 156/173 (90%) patients, with histological data on the grade of steatosis available in 147/173 (85%) patients. Histologically, an absence of liver disease was reported only in 44 (26%) of patients, while 46 (27%) patients were diagnosed with liver fibrosis, 8 (5%) with cirrhosis. In our cohort, 58 (34%) patients fulfilled MAFLD criteria and another 17 (10%) patients had steatosis without fulfilling MAFLD criteria.

Most patients underwent extensive liver resections, with 67 (39%) patients undergoing left or right hepatectomy and 92 (53%) patients undergoing extended hemi‐hepatectomy. The remaining 14 patients (8%) were treated with anatomical or nonanatomical resections or bisegmentectomies. Detailed patient and tumour characteristics are shown in Table 1. A total of 31 patients (18%) fulfilled the criteria of PHLF, while 34 patients (20%) had severe (≥Clavien‐Dindo 3b) post‐operative complications (Table S1). The 90‐day mortality was 12% (21/176) (Table S2).

**TABLE 1:** Patient and tumour characteristics of the iCCA cohort (*n* = 173).

Patient characteristics	
Age (years)	65.0 (23.0–83.0)
BMI	24.9 (13.4–41.1)
Sex ratio (F:M), *n* (%)	87 (50): 86 (50)
Aetiology by predominant histology *n* (%)	
Fibrosis	46 (27)
F1	16 (35)
F2	11 (24)
F3	7 (15)
Cirrhosis/F4	8 (5)
MAFLD	58 (34)
F0	5 (9)
F1	16 (28)
F2	9 (16)
F3	3 (5)
MAFLD‐cirrhosis /F4	4 (7)
Steatosis w/o MAFLD criteria	17 (10)
No known liver disease	44 (26)
Endoscopic biliary drainage, *n* (%)	20 (12)
Percutaneous biliary drainage, *n* (%)	4 (2)
Portal vein embolization, *n*, (%)	23 (13)
Neoadjuvant chemotherapy, *n*, (%)	16 (9)
Operative approach *n*, (%)	
Conventional	147 (85)
Laparoscopic	12 (7)
Robotic	14 (8)
Operative procedure *n*, (%)	
Atypical/anatomical resection/bisegmentectomy	14 (8)
Right hepatectomy	39 (23)
Left hepatectomy	28 (16)
Extended right hepatectomy	61 (35)
Extended left hepatectomy	31 (18)
Lymphadenectomy, *n* (%)	165 (95)
Operative time (min)	280.0 (86.0–704.0)
T category, *n* (%)	
T1	68 (39)
T2	69 (39)
T3	23 (13)
T4	13 (8)
Largest tumour diameter (mm)	60.0 (7.0–230.0)
Number of hepatic tumours	1.0 (1.0–7.0)
N category, *n* (%)	
N0	81 (47)
N1	59 (34)
R category, *n* (%)	
R0	129 (75)
R1	40 (23)
R2	1 (1)
(Micro‐) vacular invasion, *n* (%)	28 (16)
Lymphovascular invasion, *n* (%)	45 (26)
Perineural invasion, *n* (%)	35 (20)
Tumour grading, *n* (%)	
G1	6 (4)
G2	111 (64)
G3	44 (25)
Tumour stage, UICC (8th ed), *n* (%)	
I	21 (12)
II	69 (40)
III	59 (34)
IV	24 (14)
Cumulative ICU stay, days	2.0 (0.0–44.0)
Post‐operative complications, *n* (%)	
No complications	21 (12)
Clavien–Dindo I	13 (8)
Clavien–Dindo II	60 (35)
Clavien–Dindo IIIa	45 (26)
Clavien–Dindo IIIb	17 (10)
Clavien–Dindo Iva	7 (4)
Clavien–Dindo IVb	1 (1)
Clavien–Dindo V	9 (5)
Post‐operative CCI	29.6 ± (0.0–100.0)

Abbreviations: BMI, body mass index; CCI, comprehensive complication index; G, Grade; iCCA, intrahepatic cholangiocarcinoma; ICU, intensive care unit; MAFLD, metabolic dysfunction‐associated fatty liver disease; N, node; R, rest; T, tumour; UICC, Union Internationale Contre le Cancer.

*Note*: Data presented as median and range if not noted otherwise.

### 3.1 Body composition

The median period between CT imaging used for analysis and the operation was 16 days. The median BMI was 24.8 kg/m^2^ (range: 13.4–41.1 kg/m^2^), with 90 (50%) patients classified as overweight (BMI 25–30 kg/m^2^, *n* = 53, 31%) or obese (BMI ≧30 kg/m^2^, *n* = 33, 19%). Sarcopenia—deduced from a reduced SMI—was present in 30 (17%) patients, while 9 (5%) patients had a simultaneous BMI ≧25 kg/m^2^, thus fulfilling the definition of sarcopenic obesity. Visceral obesity was present in 104 (60%) patients, while 43 (25%) patients had subcutaneous obesity. Myosteatosis, reflective of reduced muscle radiodensity and fatty infiltration, was found in 45% (78/173) of the cohort. Sarcopenia was associated with higher rates of intraoperative transfusions (18/30 vs. 54/142, *p* = 0.027), with a higher rate of severe (≥Clavien‐Dindo, CD IIIb) complications (10/30 vs. 23/142, *p* = 0.030) and a higher post‐operative comprehensive complication index (CCI, 46 vs. 30, *p <* 0.001, Table S1).

### 3.2 Body composition and MAFLD

**FIGURE 1:**
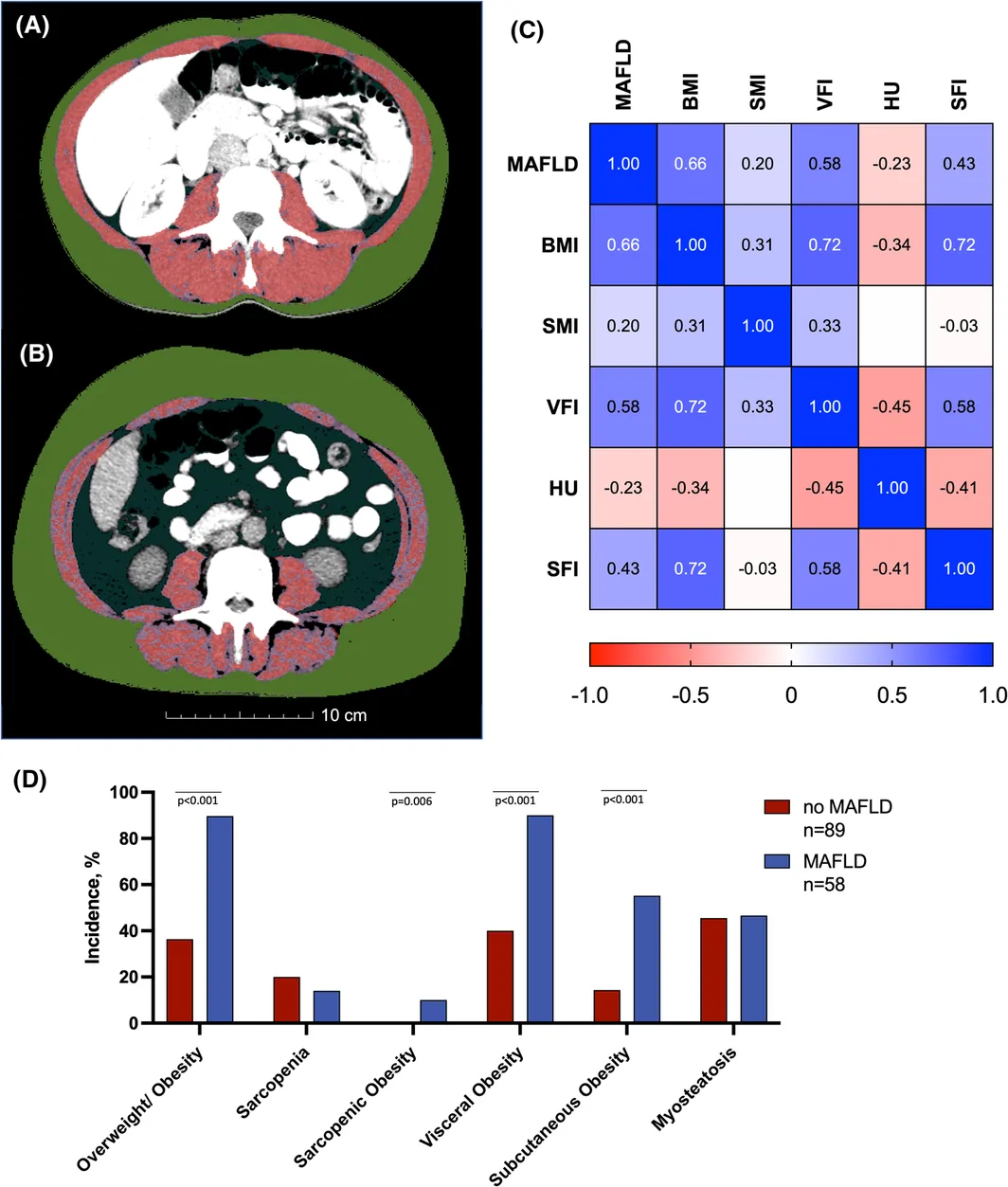
Typical physiological body composition versus typical metabolic dysfunction‐associated fatty liver disease (MAFLD)‐associated body composition. Compared to physiological body composition (A), the typical MAFLD‐associated changes of body composition depicted here (B) are an increased subcutaneous fat index (subcutaneous fat in light green), increased visceral fat area (visceral fat area, dark green/petrol), normal spinal muscle index (skeletal muscle index, red, no sarcopenia) and lower Hounsfield units‐values (grey intramuscular areas), indicative of intramuscular fat accumulation. (C) Spearman *r* correlation matrix of MAFLD and body composition parameters (*n* = 173 patients included for all columns except MAFLD [*n* = 147]) (D) Distribution of body composition pathologies between non‐MAFLD and MALFD patients with intrahepatic cholangiocarcinoma; chi‐square test with only significant values shown.

While most patient characteristics were equally distributed across body composition pathologies, MAFLD was significantly associated with pathological changes in body composition compared to without underlying liver disease, such as a significantly increased BMI (mean: 29.1 vs. 24.6 kg/m^2^, *p* < 0.001), VFI / visceral obesity (mean: 85.0 vs. 38.1 cm^2^/m^2^, *p* > 0.001) and SFI/subcutaneous obesity (mean: 79.6 vs. 58.6 cm^2^/m^2^, *p* = 0.006). Patients with predominant fibrosis/cirrhosis did not show a significantly different body composition than iCCA patients without underlying liver disease. (Table 2). When pooling all non‐MAFLD patients, and comparing their body composition to the one of patients with MAFLD, the latter had a higher median SMI (55 cm^2^/m^2^ vs. 51 cm^2^/m^2^, *p* = 0.017) and a similar incidence of sarcopenia (8/58, 14% vs. 18/89, 20% *p* = 0.591), but at the same time, a significantly higher number of patients with sarcopenic obesity was in the MAFLD group (6/58, 10% vs. 0/89, 0% *p* = 0.006). The incidence of both visceral (52/58, 90% vs. 36/89, 40% *p* < 0.001) and subcutaneous obesity (32/58, 55% vs. 13/89, 15% *p* < 0.001) was higher in the MAFLD group compared to patients without fulfilled MAFLD criteria. On average, patients with MAFLD had a lower median SM‐RA value than patients with other aetiologies (36 HU vs. 41 HU, *p* = 0.005), without translating into a higher incidence of myosteatosis due to the BMI‐adjustment of the diagnostic cut‐off (Figure 1). Furthermore, patients with MAFLD in our cohort were significantly older (median age: 67 vs. 61 years, *p* = 0.005), had a lower liver function capacity (LiMAx, Humedics GmbH) test (median LiMAx: 384 μg/kg/h vs. 462 μg/kg/h, *p* = 0.008) and a preoperatively lower CRP (median: 20 vs. 27, *p* = 0.033) than patients with other iCCA aetiologies. While prognostically important tumour‐associated factors (lymphovascular invasion, lymph node invasion) were equally distributed between patients with MAFLD and patients without liver disease, a significantly lower proportion of MAFLD patients received post‐operative chemotherapy (16/58, 39% vs. 22/44, 63%). The presence of MAFLD did not impact perioperative outcomes (Table S1). There was no significant difference in body composition across fibrosis stages F1–F4, regardless of aetiology (ANOVA, data not shown).

**TABLE 2:** Preoperative patient characteristics stratified by the main clinicopathological aetiologies.

	Entire cohort (*n* = 173)	No liver disease (*n* = 44)	MAFLD (*n* = 58)^a^	*p*=*	Fibrosis/cirrhosis (*n* = 54)	*p*=*
Age (years)	63.9 ± 11.6	62.8 ± 10.9	67.1 ± 9.9	0.186	60.9 ± 13.0	1.000
Sex ratio (F:M), *n*,(%)	87 (50): 86 (50)	24 (54): 20 (46)	26 (46): 32 (55)	0.331**	26 (48): 28 (52)	0.529**
LiMAX value (μg/kg/h)	428.3 ± 134.4	433 ± 125.4	384 ± 125.5	0.443	416. ± 132.1	1.000
PVE	23 (13)	7 (16)	10 (17)	0.698**	6 (11)	0.900**
Preoperative laboratory values						
CA19.9 (kU/L)	4213.9 ± 24172.4	850.5 ± 2938.6	6409.4 ± 30996.3	1.000	4565.2 ± 25501.4	1.000
CEA (kU/L)	7.3 ± 21.8	13.6 ± 37.2	8.4 ± 21.2	1.000	3.2 ± 2.3	0.485
Total bilirubin (mg/dL)	0.6 ± 0.7	0.7 ± 0.8	0.6 ± 0.7	1.000	0.6 ± 0.6	1.000
Albumin (g/L)	32.4 ± 16.4	32.6 ± 16.0	31.8 ± 17.8	1.000	31.7 ± 17.3	1.000
ALT (U/L)	44.7 ± 62.7	47.8 ± 83.4	43.2 ± 34.5	1.000	47.1 ± 76.9	1.000
AST (U/L)	44.2 ± 44.7	43.4 ± 49.0	39.4 ± 20.6	1.000	52.0 ± 62.3	1.000
GGT (U/L)	228.1 ± 314.6	240.9 ± 342.8	214.7 ± 396.8	1.000	249.9 ± 209.2	1.000
CRP (mg/dL)	22.2 ± 38.1	23.4 ± 27.3	19.5 ± 45.1	1.000	20.7 ± 40.9	1.000
Body composition						
BMI	25.8 ± 4.9	24.6 ± 4.6	29.1 ± 4.0	**<0.001**	24.1 ± 5.0	1.000
Female (*n* = 87)	25.1 ± 5.2	23.7 ± 4.8	27.5 ± 4.7	**<0.001**	22.8 ± 5.2	0.274
Male (*n* = 86)	26.5 ± 4.5	25.7 ± 4.2	27.8 ± 4.2	0.075	24.7 ± 5.3	0.174
Overweight/obesity (BMI ≧ 25), *n* (%)	86 (50)	16 (36)	52 (90)	**<0.001** **	17 (32)	0.611**
SMI (cm^2^/m^2^)	52.4 ± 10.5	51.5 ± 7.7	54.7 ± 10.9	0.369	52.9 ± 11.9	1.000
Female (*n* = 87)	46.7 ± 7.4	47.3 ± 6.1	46.5 ± 7.6	0.443	45.9 ± 8.2	0.364
Male (*n* = 86)	58.1 ± 10.1	56.5 ± 6.4	58.4 ± 10.6	0.556	59.0 ± 13.1	0.860
Sarcopenia, *n* (%)	30 (17)	6 (14)	8 (14)	0.928**	9 (17)	0.714**
Sarcopenic obesity, *n* (%)	9 (5)	1 (2)	6 (10)	0.117**	2 (4)	0.697**
VFI (cm^2^/m^2^)	53.7 ± 47.9	38.1 ± 32.9	85.0 ± 59.3	**<0.001**	37.9 ± 31.5	1.000
Female (*n* = 87)	46.2 ± 56.2	28.1 ± 30.4	68.5 ± 73.6	**<0.001**	28.4 ± 29.9	0.837
Male (*n* = 86)	61.3 ± 36.4	50.1 ± 32.4	80.0 ± 33.5	**0.003**	40.2 ± 32.3	0.268
Visceral Obesity, *n* (%)	104 (60)	19 (43)	52 (90)	**<0.001** **	24 (44)	0.900**
SFI (cm^2^/m^2^)	64.5 ± 34.2	58.6 ± 30.1	79.6 ± 33.9	**0.006**	56.0 ± 35.2	1.000
Female (*n* = 87)	73.6 ± 38.6	62.4 ± 33.8	88.8 ± 33.0	**0.002**	61.8 ± 45.4	0.665
Male (*n* = 86)	55.3 ± 26.4	54.0 ± 25.1	59.8 ± 28.3	0.589	48.0 ± 26.8	0.302
Subcutaneous obesity, *n* (%)	43 (25)	10 (23)	36 (62)	**0.006** **	11 (20)	0.939**
SM‐RA	38.5 ± 9.3	39.1 ± 9.6	36.1 ± 8.4	0.338	40.9 ± 9.9	1.000
Female (*n* = 87)	37.7 ± 10.0	39.6 ± 10.0	34.8 ± 8.5	0.098	40.5 ± 11.4	0.665
Male (*n* = 86)	39.3 ± 8.6	38.5 ± 9.3	38.1 ± 8.1	0.707	41.6 ± 8.1	0.392
Myosteatosis, *n* (%)	78 (45)	20 (46)	27 (47)	0.912**	22 (41)	0.639**
Risk factors/adjuvant treatment						
Lymphovascular invasion	45 (26)	12 (30)	13 (25)	0.606**	16 (36)	0.487**
Lymph node positivity	59 (34)	18 (44)	14 (30)	0.170**	17 (44)	0.978**
Adjuvant chemotherapy, *n* (%)	54 (49)	22 (63)	16 (39)	0.038**	16 (46)	0.150**
Recurrence, *n* (%)	92 (53)	28 (60)	29 (50)	0.513**	30 (60)	0.900**
Documented tumour‐related death, *n* (%)	114 (62)	29 (62)	40 (68)	0.539**	29 (58)	0.710**

Abbreviations: ALT, alanine aminotransferase; AST, aspartate aminotransferase; BMI, body mass index; CA, carbohydrate antigen; CEA, carcinoembryonic antigen; CRP, c‐reactive protein; GGT, gamma glutamyl transferase; HU, Hounsfield units; LiMAx, liver function capacity test; MAFLD, metabolic dysfunction‐associated fatty liver disease; SFI, Subcutaneous fat index; SMI, skeletal muscle index; SM‐RA, skeletal muscle radiation attenuation; VFA, visceral fat area; VFI, visceral fat index.

*Note*: Data presented as mean and standard deviation if not noted otherwise. Body composition features were defined as follows and as partly described previously^9^, ^24^: BMI – weight[kg]/height^2^[m^2^], sarcopenia—SMI < 52.4 cm^2^/m^2^. for men and <38.5 cm^2^/m^2^ for women, myosteatosis—<41 HU if BMI <25 kg/m^2^ and <33 HU if BMI ≥25 kg/m^2^, visceral obesity if VFA ≧100 cm^2^, subcutaneous obesity derived from the subcutaneous fat index, dichotomized at the upper tertile of the cohort (71.89 cm^2^/m^2^), sarcopenic obesity defined as BMI >25 kg/m^2^ and SMI ≦38.5 cm^2^/m^2^ in women and ≦52.4 cm^2^/m^2^ in men. Bold values indicate *p* values regarded as statistically significant.

^a^ Histological liver steatosis data were available in 147/173 (85%) patients.

* Based on ANOVA with post hoc Bonferroni correction, with *p* values given for comparisons to the no Liver Disease column. Bonferroni correction for three groups resulted in a level of significance *α* = 0.016.

** Based on chi‐square test, with *p* values given for comparisons to the no Liver Disease column.

### 3.3 Time to recurrence and overall survival

The median follow‐up time was 20 months postoperatively, with a median TTR of 18 months and a median OS of 28 months. During the observation period, 92 (53%) patients recurred and 113 (65%) died. The most frequent sites of recurrence were the liver (83/92, 90%), the lungs (27/92, 29%), peritoneum (22/92, 24%), lymph nodes (21/92, 23%) and the bones (16/92, 17%) with most patients experiencing simultaneous recurrence at several sites. A total of 63 (34%) patients received adjuvant chemotherapy, predominantly with gemcitabine/cisplatin or capecitabin regimens. The selection criteria for adjuvant chemotherapy in this cohort prior to the publication of the Capecitabine compared with observation in resected biliary tract cancer (BILCAP) trial in 2019^25^ were based on the presence of oncological risk factors (lymphovascular/ nodal invasion, R status) together with overall performance status.

**FIGURE 2:**
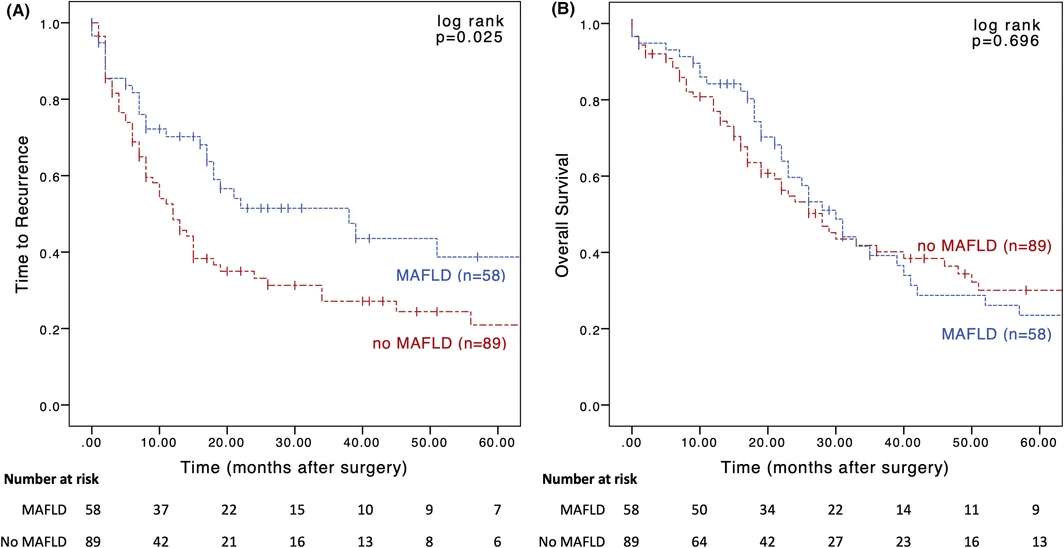
Time to recurrence (A) and overall survival (B) for patients with and without metabolic dysfunction‐associated fatty liver disease.

The most frequent histological findings in the non‐tumourous liver showed differences in TTR: patients with predominant steatosis had a trend towards longer TTR (*n* = 75, median 22 months, 95% CI: 0.1–43.9 months), compared to patients with fibrosis or no liver disease in the resected specimen (fibrosis: *n* = 44, 15 months, 95% CI: 10.4–19.6 months and no liver disease: *n* = 44, 12 months, 95% CI: 2.3–21.7 months, respectively, Figure S2, *p* = 0.127). In patients with available data on liver steatosis (*n* = 147), the MAFLD criteria were subsequently applied because patients without liver disease and without steatosis, but with fibrosis/cirrhosis grouped together in the survival analyses. Subsequently, we divided the cohort of patients with available histopathological data on steatosis into patients with MAFLD (*n* = 58) and without MAFLD (*n* = 89). A significant difference in TTR was noted between patients with MAFLD aetiology (median TTR: 38 months, 95% CI: 14.7–61.3) and patients without MAFLD (median TTR: 12 months, 95% CI: 8.0–16.0, *p* = 0.025, Figure 2). The times of OS were similar between groups.

Univariable analysis showed a significantly shorter OS of patients with myosteatosis (33 vs. 23 months, HR 1.5, log‐rank *p* = 0.020, Table 3), while other clinical variables associated with TTR and OS are listed in Table S3.

**TABLE 3:** Univariable analysis of time to recurrence and overall survival by body composition in iCCA (*n* = 173).

	*n* (%)	Median TTR (95% CI)	Hazard ratio	*p*‐Value*	Median OS (95% CI)	Hazard ratio (95% CI)	*p*‐Value*
BMI							
<25 kg/m^2^	87 (50)	14 (7.7–20.3)	‐	0.264	33 (23.0–43.0)	‐	0.350
≧25 kg/m^2^	86 (50)	22 (15.2–28.8)	‐		25 (18.6–31.4)	‐	
Sarcopenia/ reduced skeletal muscle mass							
No	142 (82)	18 (12.6–23.4)	‐	0.262	29 (24.3–33.7)	‐	0.495
Yes	30 (17)	n.a.	‐		32 (7.6–56.4)	‐	
Sarcopenic obesity							
No	163 (94)	18 (11.3–24.7)	‐	0.775	30 (24.7–35.3)	‐	0.092
Yes	9 (5)	15 (1.6–28.4)	‐		16 (5.0–27.0)	‐	
Visceral obesity							
No	69 (40)	13 (10.2–15.8)	‐	0.053	36 (14.4–57.6)	‐	0.275
Yes	104 (60)	26 (6.3–45.7)	‐		26 (20.7–31.3)	‐	
Subcutaneous obesity							
No	130 (75)	15 (9.4–20.6)	‐	0.268	31 (23.4–38.6)	‐	0.317
Yes	43 (25)	22 (14.0–30.0)	‐		23 (16.9–29.1)	‐	
Myosteatosis							
No	95 (55)	16 (11.5–20.6)	‐	0.363	33 (22.3–43.7)	1	**0.020**
Yes	78 (45)	22 (13.5–30.5)	‐		23 (18.4–27.6)	1.547 (1.065–2.246)	

Abbreviations: BMI, body mass index; CI, confidence interval; iCCA, intrahepatic cholangiocarcinoma; OS, overall survival; TTR, time to recurrence.

*Note*: Body composition features were defined as follows and as partly described previously^9^: BMI—weight [kg]/height^2^[m^2^], Sarcopenia—52.4 cm^2^/m^2^. for men and 38.5 cm^2^/m^2^ for women, Myosteatosis—<41 HU if BMI <25 kg/m^2^ and <33 HU if BMI ≥25 kg/m^2^, visceral obesity if VFA ≧100 cm^2^, subcutaneous obesity derived from the subcutaneous fat index, dichotomized at the upper tertile, sarcopenic obesity defined as BMI >25 kg/m^2^ and SMI ≦38.5 cm^2^/m^2^ in women and ≦52.4 cm^2^/m^2^ in men. Bold values indicate *p* values regarded as statistically significant.

* Based on log‐rank test.

Multivariable analysis of all significant parameters from univariable analysis showed an independent prognostic value of lymphovascular invasion, vascular invasion and lymph node invasion for TTR, while an age >65 years and fresh frozen plasma transfusions were independently prognostic for OS (Table 4). Accordingly, an independent prognostic role of MAFLD for TTR and subcutaneous obesity and myosteatosis for OS was not confirmed.

**TABLE 4:** Multivariable analysis of time to recurrence and overall survival.

	Time to recurrence (TTR)^a^		Overall survival (OS)^b^	
	Hazard ratio (95% CI)	*p*‐Value	Hazard ratio (95% CI)	*p*‐Value
MAFLD	0.622 (0.358–1.079)	0.091	‐	‐
Lymphovascular invasion	1.925 (1.076–3.445)	**0.027**	‐	‐
Vascular invasion	1.807 (1.002–3.259)	**0.049**	‐	‐
Lymph node invasion	3.912 (1.594–9.598)	**0.003**	1.260 (0.798–1.991)	0.322
Grade 3–4	1.237 (0.691–2.214)	0.475	1.314 (0.845–2.043)	0.225
UICC stage III/IV	1.403 (0.604–3.256)	0.431	‐	‐
FFP transfusions	‐	‐	2.043 (1.294–3.224)	**0.002**
R1 status	‐	‐	1.561 (0.951–2.563)	0.078
Myosteatosis	‐	‐	1.526 (0.993–2.345)	0.315
INR >1	‐	‐	1.306 (0.835–2.040)	0.715
Age >65	‐	‐	1.600 (1.036–2.471)	**0.034**

Abbreviations: CCI, comprehensive complications index; CI, confidence interval; CRP, c‐reactive protein; FFP, fresh frozen plasma; ICU, intensive care unit; INR, international normalized ratio; MAFLD, metabolic dysfunction‐associated fatty liver disease; OS, overall survival; R, rest status; TTR, time to recurrence; UICC, Union Internationale Contre le Cancer.

*Note*: Parameters with potential collinearity were excluded from the analysis. This pertains to perioperative haemoglobin (collinearity with transfusions), intraoperative blood transfusions (collinearity with FFP transfusions), CRP levels (collinearity with MAFLD aetiology), cumulative days on ICU (collinearity with CCI), adjuvant chemotherapy (patient selection in part based on prognostically relevant pathological parameters), subcutaneous obesity (collinearity with Myosteatosis), CCI (collinearity with death). Parameters that were not assumed to be stable over time (recurrence) were also excluded. Bold values indicate *p* values regarded as statistically significant.

^a^ 115 patients with complete data included.

^b^ 149 patients with complete data included.

## 4 DISCUSSION

Postoperative recurrence of iCCA with impaired long‐term survival are frequent and severe occurrences after curative‐intent surgery, despite surgical and perioperative advances in the field.^26^ In this context, due to the comparative rarity of iCCA and the challenges of curative‐intent liver resections, prognostic biomarkers are severely lacking. Recently, next‐generation sequencing revealed molecular subtypes of iCCA that carry prognostic and predictive potential,^27^ while the pathological criteria of lymph node and lymphovascular invasion are well‐characterized prognostic factors across European and Asian patient collectives.^26^, ^28^ At the same time, patient‐ or host‐centred determinants of prognosis remain relatively unexplored in this rare tumour entity.^29^ Accordingly, we investigated tumour aetiology together with body composition in a large, homogenous European cohort of patients undergoing curative‐intent liver resection for iCCA.

In this study, we were able to identify that a categorical body composition parameter —sarcopenia—was associated with perioperative outcome, but none of the body composition categories held independent prognostic value in multivariable TTR and survival analysis. As such, this study pointed towards an association of sarcopenia with perioperative morbidity, such as intraoperative transfusions, Clavien–Dindo ≥IIIb complications and an elevated post‐operative CCI. While this observation is novel to iCCA, it is shared with curative‐intent surgery for other gastrointestinal malignancies, such as HCC and pancreatic adenocarcinoma,^30^, ^31^ and is a result of malnutrition, systemic inflammation and overall catabolic processes.^32^ Furthermore, we noted inferior OS in univariable analysis in patients with myosteatosis, without observing differences when splitting the analysis by gender. Similarly, a study in a smaller palliative cohort across different CCA subtypes recently suggested a prognostic role of both myosteatosis and sarcopenia for overall survival.^33^

In the present study, a third of our cohort fulfilled the 2020 consensus MAFLD criteria.^14^ This subgroup had a higher incidence of body composition alterations, namely, sarcopenic, visceral and subcutaneous obesity, than patients without liver disease or with predominant fibrotic alterations. Recently, an association of NASH and elevated BMI as well as bioelectrical impedance analysis‐assessed fat mass and skeletal muscle mass was published in patients undergoing bariatric surgery,^8^ but data from oncological settings have not yet been reported. Because our cohort was composed of curatively treated patients without signs of systemic disease and with adequate preoperative performance status, severe cachexia, as observed in advanced gastrointestinal cancers, was less prevalent than it would be expected in palliative cohorts.

Patients with MAFLD had significantly longer TTR than patients without or with other underlying liver disease, while their OS was similar to the overall cohort. The aetiology of iCCA is relatively little studied in European/western cohorts and large meta‐analyses are oftentimes skewed towards South Asian/Pacific populations with a predominant aetiological role of *Opisthorchis viverrini*.^3^, ^34^ Across various CCA studies, the presence of cirrhosis, as well as hepatitis B and C, was not associated with shorter disease‐free survival,^34^ and the results on OS in patients with cirrhosis or chronical hepatitis B infection are disparate, with an apparent trend towards shorter OS in these patients.^35^, ^36^ To date, the vast majority of iCCA studies investigating clinical prognostic factors do not report on either body composition, steatosis, or MAFLD as an iCCA aetiology or as a prognostic factor.^34^, ^37^ Recently, an Italian study reported that patients fulfilling NASH criteria had an inferior OS after iCCA surgery but did not observe a difference in TTR between aetiologies. In fact, it remained unclear in this setting whether the accelerated mortality in the NASH group derived from cancer‐related deaths or other causes, such as increased cardiovascular mortality.^5^

The role of MAFLD as a tumour aetiology is more apparent and better characterized in HCC, for which the estimated annual incidence in patients with NASH cirrhosis is from 0.5% to 2.6%.^38^ Recent years have brought a deeper understanding of NASH‐HCC mechanisms, delineating impaired immune surveillance,^39^ autoreactive immune cells,^40^ systemic inflammation,^41^ oxidative stress due to dysregulated lipid metabolism and dysbiosis.^42^ In contrast, fatty liver disease as a contributor to iCCA has only been characterized superficially. In this context, our study contributes to a growing body of evidence that patients with MAFLD‐iCCA may constitute a distinct population for whom further metabolic, functional and immunological characteristics remain to be clarified.

The present study has a considerable sample size for European single‐centre iCCA studies, with a homogenous surgically‐treated patient collective. Nevertheless, the following shortcomings limit our conclusions: besides the exploratory single‐centre design with a retrospective evaluation of body composition and MAFLD, the patient collective had, in part, post‐operative adjuvant treatment based on negative pathological outcome factors, which was the recommended protocol before the universal recommendation for adjuvant chemotherapy from the BILCAP trial.^25^ Furthermore, we did not examine potential prognostic differences in tumour biology, such as gene variants, genetic mutations or molecular subtypes with a documented prognostic role in CCA and did not clarify their distribution across tumour aetiologies.^27^, ^43^

In conclusion, we aimed to link the complex pathophysiology of disease aetiology and body composition by investigating liver disease of iCCA patients together with CT‐derived fat and muscle parameters in the largest of these patient cohort to date. Herein, we found that MAFLD is frequent in iCCA patients, may hold potential prognostic value for time to recurrence and is significantly associated with alterations of body composition. In conjunction with the systemic changes observed in both MAFLD and in body composition pathologies, these findings illustrate the necessity of exploring systemic metabolic, performance and immunological changes in MAFLD‐iCCA patients in future studies.

## AUTHOR CONTRIBUTIONS

**Isabella Lurje:** Conceptualization (supporting); formal analysis (lead); funding acquisition (supporting); investigation (lead); methodology (supporting); visualization (supporting); writing – original draft (lead). **Deniz Uluk:** Conceptualization (equal); formal analysis (supporting); funding acquisition (supporting); project administration (equal); software (equal); supervision (supporting); writing – review and editing (equal). **Sandra Pavicevic:** Data curation (supporting); funding acquisition (supporting); project administration (supporting); writing – review and editing (equal). **Minh Duc Phan:** Conceptualization (supporting); data curation (lead); formal analysis (supporting); investigation (supporting); software (lead); visualization (equal); writing – review and editing (equal). **Dennis Eurich:** Writing – review and editing (equal). **Uli Fehrenbach:** Resources (supporting); writing – review and editing (equal). **Dominik Geisel:** Resources (supporting); writing – review and editing (equal). **Timo Alexander Auer:** Resources (supporting); writing – review and editing (equal). **Uwe Pelzer:** Resources (supporting); writing – review and editing (equal). **Dominik Paul Modest:** Writing – review and editing (equal). **Nathanael Raschzok:** Writing – review and editing (equal). **Igor Maximilian Sauer:** Resources (supporting); writing – review and editing (equal). **Wenzel Schöning:** Resources (supporting); writing – review and editing (equal). **Frank Tacke:** Funding acquisition (supporting); resources (supporting); writing – review and editing (equal). **Johann Pratschke:** Resources (supporting); writing – review and editing (equal). **Georg Lurje:** Conceptualization (equal); funding acquisition (lead); project administration (equal); resources (lead); supervision (lead); writing – review and editing (equal).

## FUNDING INFORMATION

None.

## CONFLICT OF INTEREST STATEMENT

Georg Lurje reports receiving travel support and speakers' fees from Astellas Pharma, XVIVO, Bridge to Life, Aferetica S.R.L outside the submitted work.

## ETHICS STATEMENT

The present research was conducted ethically in accordance with the World Medical Association Declaration of Helsinki. The study protocol was approved by the institute's committee on human research (Charité – Universitätsmedizin Berlin [EA1/105/21]), informed consent was waived due to the retrospective, anonymized analysis of readily available clinical data.

## Supporting information


Figure S1:

Figure S2:

Table S1:
Table S2:
Table S3:

## Data Availability

Supplementary Data is published with the manuscript. Further data can be provided upon reasonable request to the corresponding author.
