# Supplementary figures and images for: Body composition is associated with disease aetiology and prognosis in patients undergoing resection of intrahepatic cholangiocarcinoma
Source: Cancer Med. 2023 Jul 26;12(17):17569–80. doi: 10.1002/cam4.6374 (PMC10524050; doi:10.1002/cam4.6374)

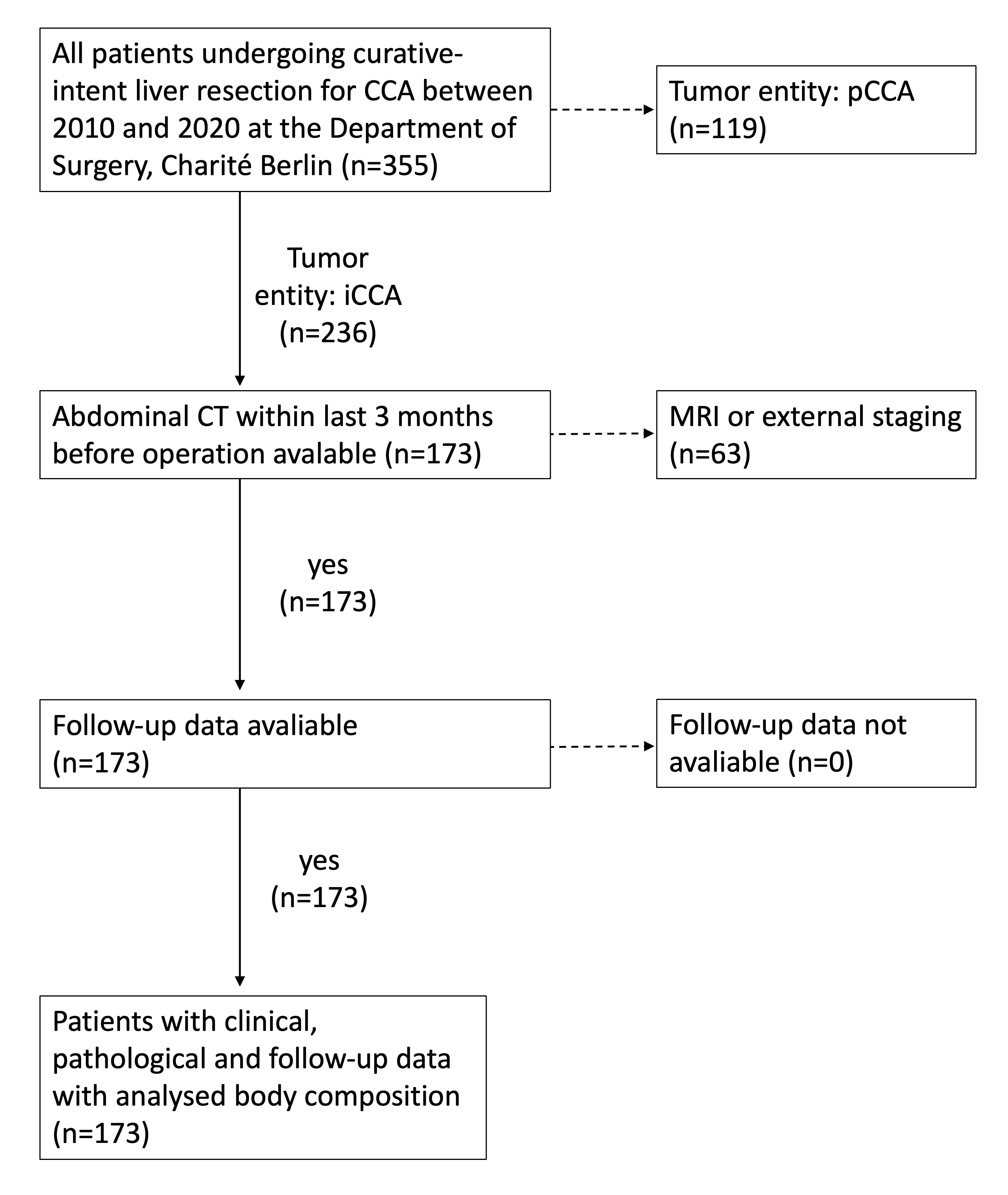

Supplement: Supplementary file 1 — Figure S1: [file CAM4-12-17569-s002.png]

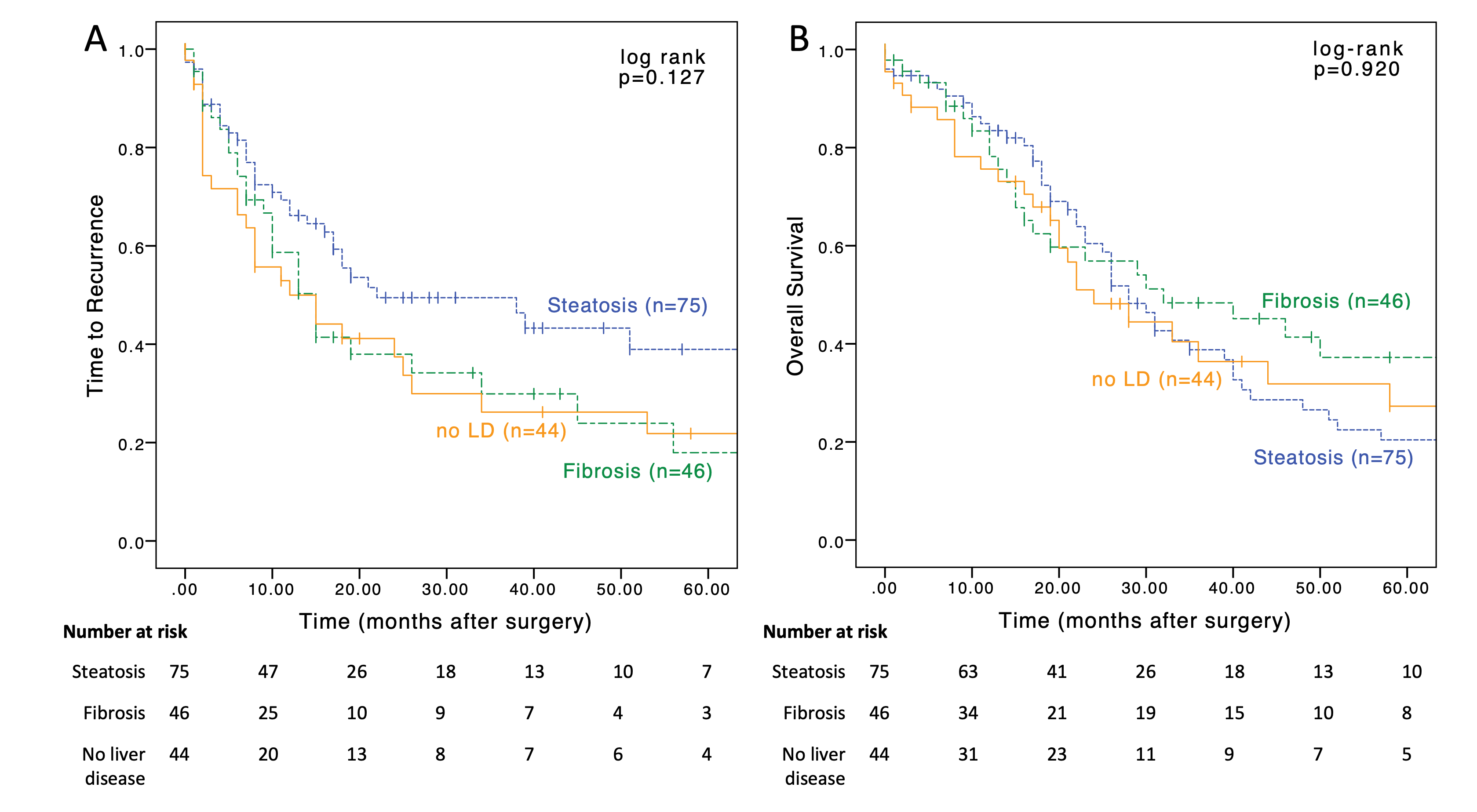

Supplement: Supplementary file 2 — Figure S2: [file CAM4-12-17569-s001.png]
