# Supplementary material for: Body composition is associated with disease aetiology and prognosis in patients undergoing resection of intrahepatic cholangiocarcinoma
Source: Cancer Med. 2023 Jul 26;12(17):17569–80. doi: 10.1002/cam4.6374 (PMC10524050; doi:10.1002/cam4.6374)
Supplement: Supplementary file 3 — Table S1: Table S2: Table S3: [file CAM4-12-17569-s003.docx]

**Supplementary Table 1**: Perioperative outcome stratified by presence of MAFLD

|  | **Entire cohort** (n=173) | **MAFLD** (n=58)^$^ | **No MAFLD** (n=89) ^$^ | p=^§^ |
| --- | --- | --- | --- | --- |
| **Intraop FFP**, n (%) | 109 (63) | 38 (66) | 55 (62) | .888^&^ |
| **Intraop Transfusions**, n (%) | 73 (42) | 22 (38) | 41 (47) | .569^&^ |
| **PHLF**, n (%) | 31 (18) | 12 (21) | 13 (15) | .489^&^ |
| **Postoperative ≥CD3b Complications**, n (%) | 34 (20) | 8 (14) | 22 (25) | .222^&^ |
| **ICU Stay** (d) | 4.83 ± 7.0 | 4.3 ± 6.4 | 5.5 ± 7.9 | .927 |
| **postoperative CCI** | 33.2 ± 23.7 | 30.1 ± 23.5 | 35.4 ± 23.9 | .099 |

Data presented as mean and standard deviation if not noted otherwise.

^§^based on Mann-Whitney U test between MAFLD and non-MAFLD columns, if not indicated otherwise

^&^based on Pearson Chi-Square Test between MAFLD and non-MAFLD columns

^$^grade of histological liver steatosis was reported in 147/173 (85%) patients

Abbreviations: CCI, comprehensive complication index; FFP, fresh frozen plasma; ICU, intensive care unit; INR, International normalized ratio; Intraop, Intraoperative; MAFLD, metabolic dysfunction-associated fatty liver disease; PHLF, post-hepatectomy liver failure; POD, postoperative day.

PHLF was defined as INR >1.15 mg/dl plus Bilirubin >1.2 mg/dl on POD 5 in patients with normal preoperative INR and Bilirubin. In patients with preoperatively elevated values and INR >1.15 mg/dl plus Bilirubin >1.2 mg/dl on POD 5, only an increase of the respective value on POD 5 compared to the previous day was considered as PHLF, according to International Study Group of Liver Surgery (ISGLS) (1). Postoperative complications were classified according to the Clavien-Dindo Score and the comprehensive complication index (2).

**Supplementary Table 2**: Association of Body composition with perioperative outcome

| Outcome | **Intrahepatic CCA (n=176)** | | | p= |
| --- | --- | --- | --- | --- |
| Reduced skeletal muscle mass (Sarcopenia) | No (n=142) | | Yes  (n=30) |  |
| Lymph node positivity  n, (%) | 49 (42) | | 10 (44) | .887 |
| Lymphovascular invasion n, (%) | 41 (33) | | 4 (15) | .060 |
| Vascular invasion,  n (%) | 26 (21) | | 2 (7) | .097 |
| Perineural invasion,  n (%) | 31 (59) | | 40 (10) | .280 |
| Intraop FFP | 88 (62) | | 21 (70) | .407 |
| Intraop Transfusions | 54 (38) | | 18 (60) | .**027** |
| PHLF | 23 (17) | | 8 (28) | .169 |
| postoperative ≥CD3b complications, n (%) | 23 (16) | | 10 (33) | .**030** |
| 90‐d mortality,  n (%) | 17 (12) | | 4 (13) | .836 |
| ICU stay (d) | 4.7±6.7 | | 5.9±8.3 | .240 |
| postoperative CCI | 30.1±22.2 | | 46.0±23.6 | **.000** |
| Myosteatosis | No (n=95) | | Yes (n=78) |  |
| Lymph node positivity  n, (%) | 36 (45) | | 23 (38) | .429 |
| Lymphovascular invasion n, (%) | 22 (28) | | 23 (31) | .698 |
| Vascular invasion,  n (%) | 18 (22) | | 10 (13) | .170 |
| Perineural invasion,  n (%) | 19 (58) | | 16 (53) | .735 |
| Intraop FFP | 56 (59) | | 53 (68) | .222 |
| Intraop Transfusions | 38 (40) | | 35 (45) | .519 |
| PHLF | 17 (19) | | 14 (19) | .975 |
| postoperative ≥CD3b complications, n (%) | 14 (15) | | 20 (26) | .073 |
| 90‐d mortality,  n (%) | 13 (14) | | 9 (12) | .673 |
| ICU stay (d) | 4.1±5.8 | | 5.7±8.1 | .211 |
| postoperative CCI | 30.2±22.7 | | 36.9±24.5 | .095 |
| Subcutaneous obesity | Lower two tertiles (n=98) | | Upper tertile (n=43) |  |
| Lymph node positivity  n, (%) | 46 (47) | | 13 (30) | .064 |
| Lymphovascular invasion n, (%) | 32 (31) | | 14 (28) | .698 |
| Vascular invasion  n,(%) | 21 (20) | | 7 (14) | .351 |
| Perineural invasion,  n (%) | 27 (59) | | 9 (50) | .528 |
| Intraop FFP | 74 (64) | | 36 (62) | .824 |
| Intraop Transfusions | 52 (45) | | 22 (38) | .386 |
| PHLF  n (%) | 19 (17) | | 12 (22) | .433 |
| 90‐d ≥CD3b complications, n (%) | 22 (19) | | 12 (21) | .787 |
| 90‐d mortality,  n (%) | 12 (10) | | 10 (17) | .197 |
| ICU stay (d) | 4.8±6.93 | | 4.9±7.3 | .795 |
| postoperative CCI | 33.2±23.6 | | 33.7±26.8 | .682 |
| Visceral Obesity | | No (n=69) | Yes (n=104) |  |
| Lymph node positivity  n, (%) | 28 (45) | | 31 (40) | .519 |
| Lymphovascular invasion n, (%) | 19 (32) | | 26 (28) | .653 |
| Vascular invasion  n, (%) | 12 (19) | | 16 (17) | .710 |
| Perineural invasion,  n (%) | 15 (58) | | 20 (54) | .775 |
| Intraop FFP | 35 (51) | | 74 (71) | **.006** |
| Intraop Transfusions | 26 (38) | | 47 (45) | .327 |
| PHLF  n (%) | 12 (17) | | 22 (21) | .542 |
| 90‐d ≥CD3b complications, n (%) | 12 (18) | | 23 (21) | .555 |
| 90‐d mortality,  n (%) | 11 (16) | | 11 (11) | .300 |
| ICU stay (d) | 4.7±5.9 | | 4.9±7.6 | .498 |
| postoperative CCI | 33.2±24.9 | | 33.3±22.9 | .890 |
| Sarcopenic Obesity | No (n=163) | | Yes (n=9) |  |
| Lymph node positivity  n, (%) | 57 (43) | | 2 (33) | .655 |
| Lymphovascular invasion n, (%) | 44 (31) | | 1 (11) | .206 |
| Vascular invasion,  n (%) | 27 (19) | | 1 (11) | .576 |
| Perineural invasion,  n (%) | 33 (55) | | 2 (67) | .691 |
| Intraop FFP | 101 (62) | | 8 (89) | .103 |
| Intraop Transfusions | 67 (41) | | 5 (56) | .392 |
| PHLF  n (%) | 27 (17) | | 4 (44) | **.040** |
| 90‐d ≥CD3b complications, n (%) | 30 (18) | | 3 (33) | .268 |
| 90‐d mortality,  n (%) | 20 (12) | | 1 (11) | .918 |
| ICU stay (d) | 4.95±7.1 | | 4.67±3.0 | .317 |
| postoperative CCI | 32.1±22.7 | | 46.9±28.6 | .074 |
| BMI | No (n=89) | | Yes (n=87) |  |
| Lymph node positivity  n, (%) | 35 (45) | | 24 (36) | .147 |
| Lymphovascular invasion n, (%) | 25 (33) | | 20 (26) | .374 |
| Vascular invasion,  n (%) | 17 (21) | | 11 (14) | .239 |
| Perineural invasion,  n (%) | 19 (58) | | 16 (53) | .735 |
| Intraop FFP | 52 (60) | | 57 (66) | .375 |
| Intraop Transfusions | 40 (46) | | 33 (38) | .311 |
| PHLF  n (%) | 14 (17) | | 17 (21) | .526 |
| 90‐d ≥CD3b complications, n (%) | 18 (21) | | 16 (19) | .730 |
| 90‐d mortality,  n (%) | 11 (13) | | 11 (13) | .977 |
| ICU stay (d) | 4.8±7.0 | | 4.8±7.0 | .813 |
| 90‐d CCI | 34.4±22.4 | | 32.1±24.8 | .345 |

Data presented as mean ± Standard deviation if not noted otherwise

For continuous variables, Mann-Whitney U Test was applied, Chi Square Test or Fisher’s exact test were employed for categorical variables.

Abbreviations: BMI, body mass index; CCI, comprehensive complication index; FFP, fresh frozen plasma; ICU, intensive care unit; PHLF, post-hepatectomy liver failure; POD, postoperative day.

PHLF was defined as: INR >1.15 mg/dl plus Bilirubin >1.2 mg/dl on POD 5 in patients with normal preoperative INR and Bilirubin. In patients with preoperatively elevated values and INR >1.15 mg/dl plus Bilirubin >1.2 mg/dl on POD 5, only an increase of the respective value on POD 5 compared to the previous day was considered as PHLF.

**Supplementary Table 3**: Univariable analysis of disease-free and overall survival by clinico-pathological characteristics

|  | **n (%)** | **Median DFS (95%CI)** | **Hazard Ratio** | | **p** † | **Median OS (95%CI)** | **Hazard Ratio** | **p** † |
| --- | --- | --- | --- | --- | --- | --- | --- | --- |
| **Sex** |  |  |  | |  |  |  |  |
| Male | 86 (50) | 19 (11.8-26.2) |  | | .920 | 28 (15.9-40.1) |  | .337 |
| Female | 87 (50) | 18 (6.2-29.7) |  | |  | 30 (24.3-35.7) |  |  |
| **Age** (years) |  |  |  | |  |  |  |  |
| ≤ 65 | 91 (53) | 15 (8.2-21.8) |  | | .371 | 36 (24.3-47.7) | 1 | **.031** |
| > 65 | 82 (47) | 22 (14.0-30.0) |  | |  | 23 (16.5-29.5) | 1.506 (1.032-2.199) |  |
| **PVE** |  |  |  | |  |  |  |  |
| No | 150 (87) | 19 (11.0-27.1) |  | | .053 | 30 (24.8-35.2) |  | .218 |
| Yes | 23 (13) | 13 (3.6-22.4) |  | |  | 19 (17.4-20.6) |  |  |
| **Total Bilirubin,** mg/dl | |  |  | |  |  |  |  |
| ≤ 1.2 | 154 (89) | 18 (10.8-25.2) |  | | .170 | 28 (22.0-34.0) |  | .318 |
| > 1.2 | 11 (6) | 15 (0.0-30.2) |  | |  | 33 (5.2-60.8) |  |  |
| **Albumin,** g/l |  |  |  | |  |  |  |  |
| ≤ 42 | 63 (36) | 25 (16.4-33.6) |  | | .738 | 23 (17.1-29.0) |  | .074 |
| > 42 | 44 (25) | 21 (0.0-44.5) |  | |  | 44 (30.4-57.6) |  |  |
| **AST,** U/l |  |  |  | |  |  |  |  |
| ≤ 40 | 113 (65) | 18 (2.1-33.9) |  | | .371 | 28 (21.1-34.9) |  | .424 |
| > 40 | 54 (31) | 19 (6.2-31.8) |  | |  | 25 (4.2-45.8) |  |  |
| **ALT,** U/l |  |  |  | |  |  |  |  |
| ≤ 40 | 108 (62) | 16 (9.4-22.6) |  | | .403 | 28 (21.4-34.6) |  | .897 |
| > 40 | 43 (25) | 26 (0.5-51.5) |  | |  | 36 (15.0-56.9) |  |  |
| **GGT,** U/l |  |  |  | |  |  |  |  |
| ≤ 100 | 75 (43) | 17 (0.0-35.5) |  | | .617 | 28 (17.7-38.3) |  | .360 |
| > 100 | 90 (52) | 18 (9.6-26.4) |  | |  | 28 (20.7-35.3) |  |  |
| **INR** |  |  |  | |  |  |  |  |
| ≤ 1 | 78 (45) | 17 (13.4-20.6) |  | | .737 | 31 (7.1-54.9) | 1 | **.035** |
| > 1 | 93 (54) | 22 (4.0-40.0) |  | |  | 26 (19.4-32.6) | 1.500  (1.023-2.200) |  |
| **Hemoglobin,** g/dl | | |  | |  |  |  |  |
| ≤ 12 | 38 (22) | 16 (7.2-24.8) |  | .222 | | 20 (14.6-25.5) | 2.064  (1.372-3.205) | **.000** |
| > 12 | 65 (38) | 19 (11.6-26.4) |  | |  | 35 (25.1-44.9) | 1 |  |
| **CRP,** mg/l |  |  |  | |  |  |  |  |
| ≤ 10 | 42 (24) | 25 (8.8-41.2) | 1 | | **.004** | 30 (23.9-36.1) |  | .121 |
| > 10 | 61 (35) | 10 (4.2-15.8) | 2.073 (1.241-3.464) | |  | 20 (16.2-23.8) |  |  |
| **Intraoperative blood transfusions** | | |  | |  |  |  |  |
| No | 100 (58) | 19 (10.4-27.6) |  | | .722 | 33 (23.9-42.1) | 1 | **.025** |
| Yes | 73 (42) | 18 (6.2-29.8) |  | |  | 12 (15.4-30.6) | 1.519  (1.049-2.201) |  |
| **Intraoperative FFP transfusions** | | | | |  |  |  |  |
| No | 64 (37) | 34 (15.2-52.8) |  | | .133 | 52 (19.4-84.6) | 1 | **.000** |
| Yes | 109 (63) | 16 (10.4-21.6) |  | |  | 23 (17.2-28.8) | 2.117  (1.390-3.225) |  |
| **R status** |  |  |  | |  |  |  |  |
| R0 | 129 (75) | 21 (7.3-34.7) |  | | .133 | 31 (22.0-40.0) | 1 | **.000** |
| R1/Rx | 40 (23) | 13 (7.7-18.3) |  | |  | 24 (16.7-31.3) | 1.436  (0.947-2.177) |  |
| **Microvascular Invasion** | | |  | |  |  |  |  |
| No | 126 (73) | 19 (11.8-26.2) | 1 | | **.011** | 30 (24.2-35.8) |  | .279 |
| Yes | 27 (16) | 10 (6.6-13.4) | 1.852  (1.130-3.034) | |  | 22 (17.5-26.6) |  |  |
| **Perineural invasion** | |  |  | |  |  |  |  |
| Pn0 | 27 (16) | 13 (9.2-16.8) |  | | .644 | 26 (n.a.) |  | .772 |
| Pn1 | 35 (20) | 18 (12.1-24.0) |  | |  | 30 (19.9-40.1) |  |  |
| **Lymphovascular invasion** | |  |  | |  |  |  |  |
| No | 107 (62) | 26 (11.5-40.5) | 1 | | **.000** | 31 (23.3-38.7) |  | .073 |
| Yes | 45 (20) | 10 (6.2-13.8) | 2.147  (1.374-3.355) | |  | 19 (15.3-22.7) |  |  |
| **Tumor grading** |  |  |  | |  |  |  |  |
| G1 / G2 | 114 (66) | 26 (8.9-43.0) | 1 | | .**030** | 32 (21.3-42.7) | 1 | **.046** |
| G3 / G4 | 44 (25) | 12 (7.3-16.7) | 1.657  (1.038-2.644) | |  | 21 (14.2-27.8) | 1.607  (1.035-2.496) |  |
| **Tumor stage (UICC)** | | |  | |  |  |  |  |
| I / II | 87 (50) | 38 (4.4-71.6) | 1 | | **.009** | 39 (27.2-50.8) |  | .053 |
| III /IV | 82 (47) | 13 (8.5-17.4) | 1.688  (1.114-2.558) | |  | 23 (14.8-31.2) |  |  |
| **pT stage** |  |  |  | |  |  |  |  |
| pT1-2 | 137 (79) | 17 (10.2-23.8) |  | | .406 | 31 (23.1-38.9) |  | .155 |
| pT3-4 | 36 (21) | 26 (8.2-43.8) |  | |  | 16 (2.4-29.5) |  |  |
| **Nodal invasion** |  |  |  | |  |  |  |  |
| pN0 | 81 (47) | 34 (14.0-54.0) | 1 | | **.000** | 39 (25.9-52.1) | 1 | **.004** |
| pN1 | 59 (34) | 10 (6.8-13.2) | 2.697  (1.691-4.302) | |  | 18 (12.3-23.7) | 1.607  (1.035-2.496) |  |
| **Cumulative ICU stay, days** | | |  | |  |  |  |  |
| Mean ± SD | 4.8 ± 7.0 |  |  | | .930 |  | 1.030 (1.005-1.055) | **.000** |
| **CCI** |  |  |  | |  |  |  |  |
| ≤ 40 | 115 (67) | 22 (9.1-34.9) |  | | .220 | 36 (24.4-47.6) | 1 | **.000** |
| > 40 | 54 (31) | 15 (9.8-20.2) |  | |  | 17 (11.5-22.5) | 2.284 (1.523-3.424) |  |
| **Adjuvant chemotherapy** | | | | |  |  |  |  |
| No | 53 (31) | 26 (5.3-46.7) | 1 | | **.016** | 57 (27.1-86.9) |  | **.000** |
| Yes | 59 (34) | 12 (7.7-16.3) | 1.496 (0.927-2.415) | |  | 28 (19.2-36.8) |  |  |
| **Postoperative recurrence** | | |  | |  |  |  |  |
| No | 81 (47) |  |  | |  | 57 (5.5-108.5) | 1 | **.025** |
| Yes | 92 (53) |  |  | |  | 28 (21.7-34.3) | 1.962  (1.070-3.596) |  |

^†^Based on log rank test, for continuous variables (ICU stay, Hospital stay, based on Cox regression analysis)

Abbreviations: ALT, alanine aminotransferase; AST, aspartate aminotransferase; BMI, body mass index; CCI, comprehensive complication index; CI, confidence interval; CRP, c-reactive protein; DFS, disease-free survival; FFP, fresh frozen plasma; GGT, gamma glutamyl transferase; ICU, intensive care unit; INR, international normalized ratio; OS, overall survival; pT, pathological Tumor stage; PVE, portal vein embolization; R, rest; UICC, Union internationale contre le cancer.

1. Rahbari NN, Garden OJ, Padbury R, Brooke-Smith M, Crawford M, Adam R, et al. Posthepatectomy liver failure: a definition and grading by the International Study Group of Liver Surgery (ISGLS). *Surgery* (2011) 149(5):713-24. Epub 2011/01/18. doi: 10.1016/j.surg.2010.10.001. PubMed PMID: 21236455.

2. Slankamenac K, Graf R, Barkun J, Puhan MA, Clavien PA. The comprehensive complication index: a novel continuous scale to measure surgical morbidity. *Annals of surgery* (2013) 258(1):1-7. doi: 10.1097/SLA.0b013e318296c732. PubMed PMID: 23728278.
